# Effect of microstructure refinement of pure copper on improving the performance of electrodes in electro discharge machining (EDM)

**DOI:** 10.1038/s41598-023-43584-y

**Published:** 2023-10-04

**Authors:** Jacek Skiba, Mariusz Kulczyk, Sylwia Przybysz-Gloc, Monika Skorupska, Mariusz Kobus, Kamil Nowak

**Affiliations:** 1 Institute of High Pressure Physics, Polish Academy of Sciences (Unipress), ul. Sokołowska 29, 01-142 Warszawa, Poland; 2 Gemet Elżbieta Czerwieniak, ul. Lisia 16, 05-410 Józefów, Poland

**Keywords:** Mechanical engineering, Metals and alloys, Mechanical properties, Design, synthesis and processing

## Abstract

The paper presents an analysis of the impact of plastic deformation using hydrostatic extrusion (HE) on the structural, mechanical and functional properties of pure copper for use as electrodes in the process of electro discharge machining (EDM). As part of the research, copper was subjected to the HE process with the maximum cumulative true strain equal to *ɛ*_*cum*_ = 3.89 obtained in 5 stages. The result was, a refinement of the microstructure with the grains elongated in the direction of extrusion, with a cross-sectional size of *d*_2_ = 228 nm. As the obtained material can be potentially used in the process of electro discharge machining, the copper specimens after the HE process were subjected to a comprehensive analysis to determine the mechanical, physical and functional properties of the material. A significant increase in strength (*UTS*) and yield strength (*YS*) of the HE-processed copper was obtained, reaching respectively *UTS* = 464 MPa and *YS* = 456 MPa at the maximum strain of *ɛ* = 3.89. Despite the clear strain-induced strengthening of the material, a very high electrical conductivity of not less than 97% was obtained. The electrodes made of copper after HE process have reduced erosion wear while maintaining a comparable or better quality of the machined surface. The best results were obtained for finish machining, where the electrical discharge wear was lower by 60% compared to the electrode made of non-processed copper. In addition, an improvement in the surface quality after the EDM process by 25% was observed when using the HE-processed electrodes.

## Introduction

The electrical discharge machining (EDM) process is one of the nonconventional methods of machining. This process was developed around 1943^1^. In the EDM process, the material is removed by the phenomena accompanying electrical discharges occurring in the area of the inter-electrode gap. This gap is an empty area between the workpiece and the working tool electrode. The energy transferred during the discharge in the form of a spark causes the metal to melt and evaporate. The only but the key limitation of the EDM method of machining is the need to conduct electric current through the machined material. An undoubted advantage over other methods of machining is the fact that the hardness of the workpiece is not a technological limitation.

As a result, this method of machining can be successfully used for the production of parts from state-of-the-art materials in sectors such as aviation, automotive and medicine. In addition, due to the high accuracy of machined surface (approx. 5 μm), this process is also suitable for the production of micro-shapes, including micro-holes in the diameter range of 1–999 μm^2,3^.

Regardless of the target application of EDM machining, technical development forces crossing more and more quality boundaries resulting from the use of increasingly complex shapes of the machined components. As a result, efforts are still being made to improve the performance of the process to increase the parameters and accuracy of the manufactured shapes.

The cost of an EDM-produced part is basically determined by the cost of the electrode, which consists of the cost of the raw material plus the cost of its manufacturing. The cost of the electrode used can be 70% of the entire process cost. The production of electrodes and the EDM machining time constitute the major cost of the process—they both can account for more than 50% of conventional machining costs^4^.

The thermophysical properties of the electrode, such as thermal and electrical conductivity and thermal expansion, have a significant impact on the efficiency of the EDM process^5^.

Therefore, when selecting the material for the electrode, all the properties affecting the EDM process should be taken into consideration.

Since the EDM process is based on the electric discharge phenomenon at the interface between the tool (electrode) and the machined material, it is obvious that the electrical properties of the electrode used have a key impact on the EDM machining process. Higher electrical conductivity (low electrical resistivity) translates into slower wear of the tool.

In addition, although the EDM process does not involve mechanical forces, the generated sparks act violently causing microscopies stress in the material^6^. Thus, the strength, structural integrity and homogeneity of the electrode are critical factors influencing the efficiency of the EDM machining process and the operating life of the electrode.

Another important factor when selecting the electrode material is its machinability, which can significantly affect the cost of electrode production. If the selected material is too difficult to machine or its cost is very high, it is not suitable for electrical discharge machining and its applications are very limited^5^.

Copper is the most commonly used material for EDM electrodes due to its high electrical and thermal conductivity. This is confirmed by numerous publications by Czeluśniak et al.^7^. Copper also has good structural integrity and machinability. However, the copper electrode has some disadvantages, like for example, high volumetric expansion and low melting point, which may adversely affect the process of “washing out” the particles from the working gap and, consequently, accelerate the wear of the electrode^8^. Another disadvantage of copper is that it is a ductile material, which makes it difficult to obtain the appropriate quality of the electrode surface by machining^9^.

In order to improve the machinability of copper, alloy admixtures are used, such as, for example, tellurium, but they have a negative effect on the wear of the electrode compared to pure copper^6^. The addition of tellurium to copper improves its machinability but accelerates its wear and reduces the material compared to pure copper.

Due to the aforementioned disadvantages and limitations versus pure copper as a material for electrodes for the EDM process, intensive research projects are being conducted to find appropriate materials to increase the efficiency of the process. The materials used for the electrodes include copper and its alloys^10–12,20^, graphite^13,14^, copper graphite^15^, tellurium copper^6^, tungsten^16,20^, tungsten carbide^16^, silver^17^, silver tungsten^18^ and aluminum^19,20^.

The authors of the present paper have attempted to use an unconventional method of plastic deformation by hydrostatic extrusion (HE) in order to improve the functional properties of pure copper used in the production of electrodes for EDM machining.

Hydrostatic extrusion, thanks to the triaxial compressive stresses in the strain zone and the favourable lubrication conditions during the process of extrusion, allows effective deformation of materials with large unit strains while maintaining the structural and mechanical homogeneity of the extruded material throughout its entire volume. The research that has been conducted at the Institute of High Pressure Physics, Polish Academy of Sciences Unipress IHPP PAS for 45 years has confirmed the exceptional suitability of HE for generating severe plastic strains in materials, including materials that cannot be strained with the use of conventional methods such as rolling, drawing or conventional extrusion. This allows to give the materials new, higher strength^21–24^, fatigue^25^, impact strength^26^, tribological^27^ and corrosion^28^ properties, while improving functional properties such as machinability^29,30^ or electrical conductivity^31^.

In this paper, the authors attempted to analyse the effect of microstructure refinement of pure copper obtained by hydrostatic extrusion on the performance properties of electrodes used in the EDM process. The change of basic material properties such as strength, yield strength and electrical conductivity has a direct impact on technological processes, including EDM processes. Electrodes made of copper with higher parameters are characterized by higher stiffness and dimensional stability during machining, and show less EDM wear while maintaining better quality of the machined surface compared to electrodes made of undeformed copper by HE.

Many publications on the modification of the microstructure and properties of materials after large plastic deformation indicate their potential application. However, limitations related to the geometry of the resulting products or surface quality often prevent their practical use. This is especially true for frequently cited processes such as Equal Channel Angular Pressing (ECAP) or High Pressure Torsion (HPT). The HE process offers the possibility of real use of plastically processed materials in industry. Studies, related to the viability of the final products, which are EDM electrodes made of ultrafine-grained copper, confirm this fact and undoubtedly constitute an element of novelty in this publication.

The results analysed in this paper directly allow the market implementation of a new unique product—electrodes for the EDM process of ultrafine copper.In the era of intensively developing unconventional manufacturing and material modification techniques, it is necessary to analyse their performance properties in detail in order to realize their full application potential.

## Material and experimental methods

The basic properties of the tested copper in the initial state are shown in Table 1 below was tested.

**Table 1:** Properties of copper 99.95% in the initial state.

Material	Tensile strength, *UTS* (MPa)	Yield strength, *YS* (MPa)	Elongation, *A* (%)	Hardness, *HV*_0*.*2_	Electrical conductivity, *IACS* (%)
Cu 99.95%	258	246	23	101	100

The material was subjected to the process of plastic deformation by hydrostatic extrusion in two processing variants:

- one-stage extrusion—including deformation with three different degrees of strain in the range from ɛ = 1.12–2.53.
- cumulative extrusion—involving multiple deformations of the material with increasingly smaller degrees of strain with cumulative strain after 5 stages of HE equal to ɛ_cum_ = 3.89.

The HE hydrostatic extrusion process was carried out on presses designed and manufactured at IHPP PAS Unipress with working pressures up to 1800 MPa, equipped with a system of cooling the extruded product with running cold water in order to minimise the effect of adiabatic heating.

The microstructure observations in the initial state of the material were carried out using a Nikon Eclipse LV150 LM light microscope, while the microstructure observations after the HE process were carried out using a TEM JEOL 1200 EX transmission electron microscope. In both cases, observations were made on the cross-section of the extruded round rod. Grain sizes were quantified using "Mikrometr" software^32^. The data was based on TEM images. After imaging and mapping of at least 200 grains randomly selected from the set, the equivalent diameter *d*_2_ was calculated. The tests of mechanical properties were carried out using a Zwick-Roell Z250 testing machine with a maximum force of 250 kN to determine the tensile strength (*UTS*), yield point (*YS*) and elongation to break *ε*_*f*_*.* The tests were carried out in accordance with PN-EN ISO 6892-1 at a tensile speed of 0.008 s^−1^ on fivefold specimens with a diameter of 6 mm, drawn along the axis of the bars. Microhardness measurements were made on the cross-section of the extruded bars using a Zwick-Roell ZHV1-A hardness tester under a load of 200 g for 15 s.

Electrical conductivity tests (%*IACS*) were performed using a SIGMATEST 2.069-Forester instrument. The tests were carried out on the cross-section and the longitudinal section of the copper samples after the HE process.

**Figure 1:**
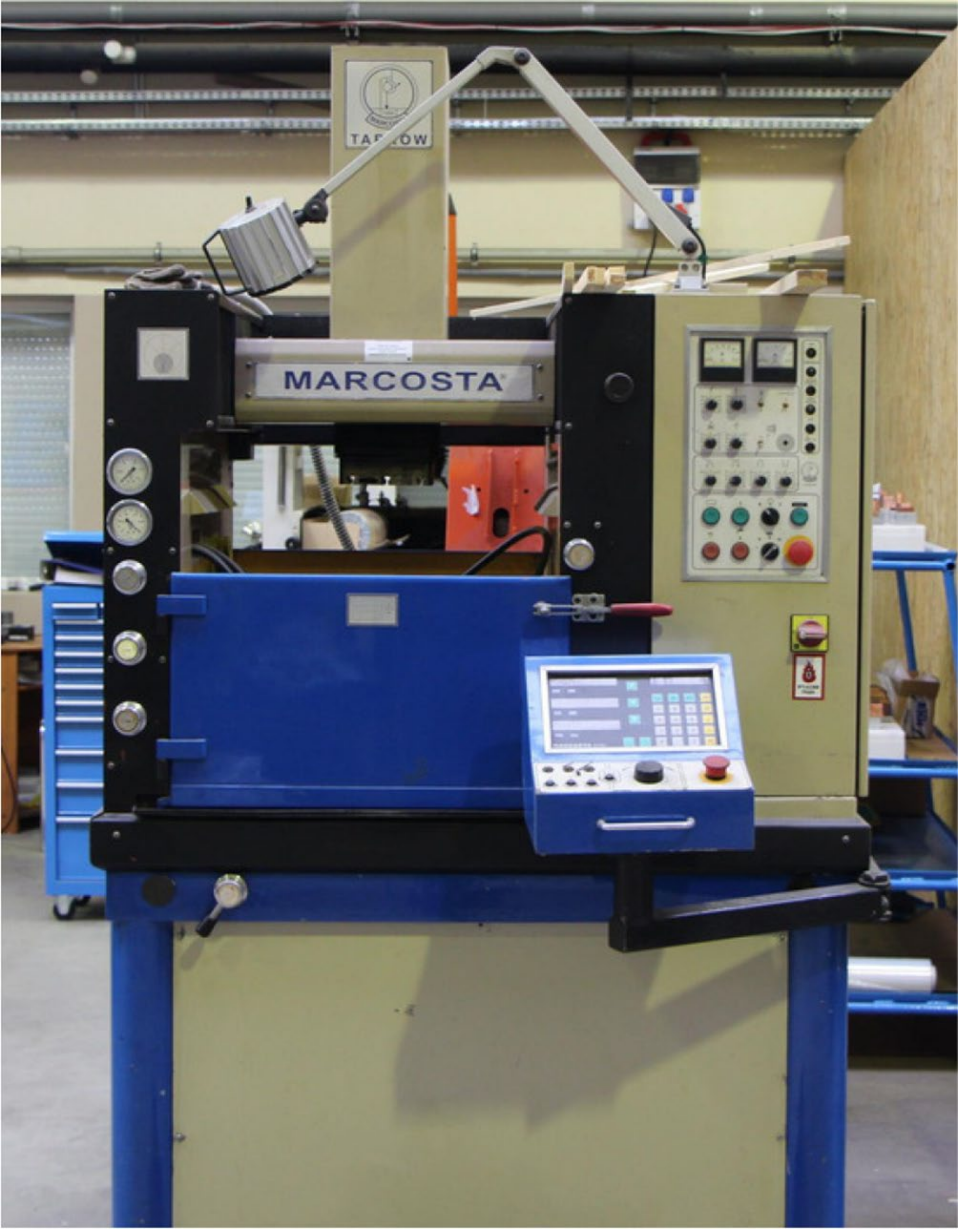
Wear test bench for the EDM process.

The tested material was used to make electrodes for the electro discharge machining process—cylinders with a diameter of 10 mm and a height of 60 mm. EDM machining operation was carried out using the laboratory ZAP EDMA-40, Fig. 1, in two machining variants involving extreme machine settings allowing the process to be tested under conditions corresponding to roughing and finishing. Detailed parameters of the EDM process are shown in Table 2. EDM wear was estimated based on measurements of the height of the working part of the electrode before and after the EDM process. The measurements were carried out using precision measuring devices such as calipers and micrometers that provide a measurement accuracy of 0.01 mm.

**Table 2:** EDM machining parameters in wear tests.

Type of machining	Working amperage *I*_*r*_ (A)	Pulse time *t* (ms)	Depth of machining *H* (mm)
Rough	12	9	20
Finish	3	4	20

## Results and discussion

### Hydrostatic extrusion

The HE process parameters are shown in Table 3 below.

**Table 3:** Basic parameters of the hydrostatic extrusion process for copper 99.9%.

Specimen	Initial diameter, *d*_0_ (mm)	Product diameter, *d*_*f*_ (mm)	True strain, *ɛ* = lnR^(a)^	Cumulative true strain, *ɛ*_*cum*_	Adiabatic temperature, *T* (°C)	*T/T*_*m*_^(b)^	Hydrostatic extrusion pressure, *p*_*HE*_ (MPa)
1 × HE							
1CuT	69.87	39.87	1.12	1.12	119	0.29	480
2CuT	69.87	29.74	1.71	1.71	172	0.33	695
3CuT	69.87	19.76	2.53	2.53	252	0.39	1016
5 × HE							
1CuT	69.87	39.87	1.12	1.12	119	0.29	480
1CuT-2	39.87	29.86	0.58	1.70	114	0.29	377
1CuT-3	29.86	19.90	0.81	2.51	125	0.29	460
1CuT-4	19.90	15.00	0.57	3.08	139	0.30	503
1CuT-5	15.00	9.98	0.81	3.89	119	0.29	561

^(a)^R* –* reduction ratio = initial to final cross section.

^(b)^*T*_*m*_ – melting point = 1083 °C.

**Figure 2:**
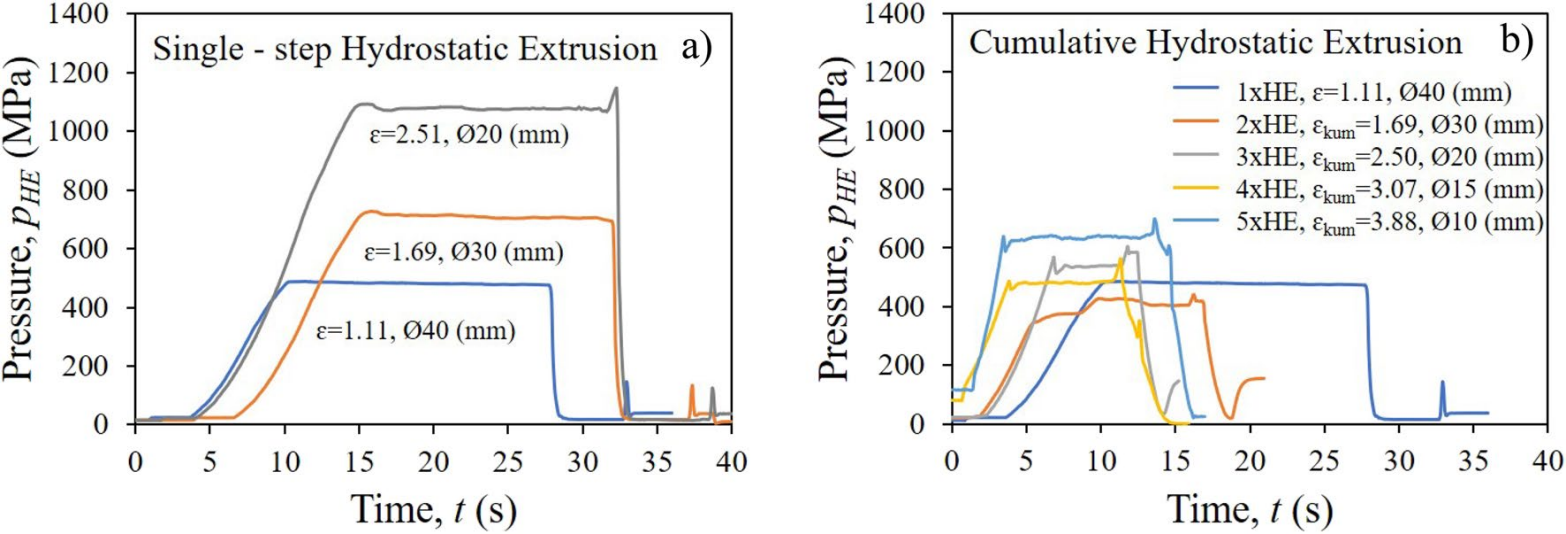
Pressure characteristic of hydrostatic extrusion of copper: (**a**) single-step HE, (**b**) cumulative HE.

Each stage of the HE process showed stable, almost linear extrusion characteristics, which confirms the correctness of the adopted technological parameters, like the geometry of tools and the deformation rate, Fig. 2.

Although the HE process was carried out under conditions of intensive cooling of the extruded product, the adiabatic temperature generated in the HE process was estimated directly in the deformation zone during the test, and the obtained values for individual stages are given in Table 3.

In works describing the HE process, the effect of strong adiabatic heating generated during the process has been noted multiple times^33–35^. It results from the mechanical work of the plastic strain performed during the process of extrusion and its conversion into heat. As presented, e.g. in^35^, the effect of strong adiabatic heating is directly proportional to the extrusion pressure and inversely proportional to the density and specific heat of the material. It also depends on the amount of work converted into heat, which in the case of HE is very high and can reach up to 95%. The calculated value of the adiabatic heating effect in this work indicates the values *T/T*_*m*_ ~ 0.3 that are equal for the cumulative process, while for the one-stage process with the maximum unit strain equal to ɛ = 2.53, the value of *T/T*_*m*_ is almost 0.4, Table 3, where *T* is the temperature measured during the process of extrusion and *T*_*m*_ is the melting point of the material, both in K. The obtained values of homologous temperatures in both variants of strain may indicate more intensive healing and recrystallisation processes occurring in the one-stage process, while in the case of cumulative extrusion, where the homologous temperature is much lower, we can expect much more effective strain strengthening of the material resulting from the accumulation of structural defects.

### Microstructure evaluation

**Figure 3:**
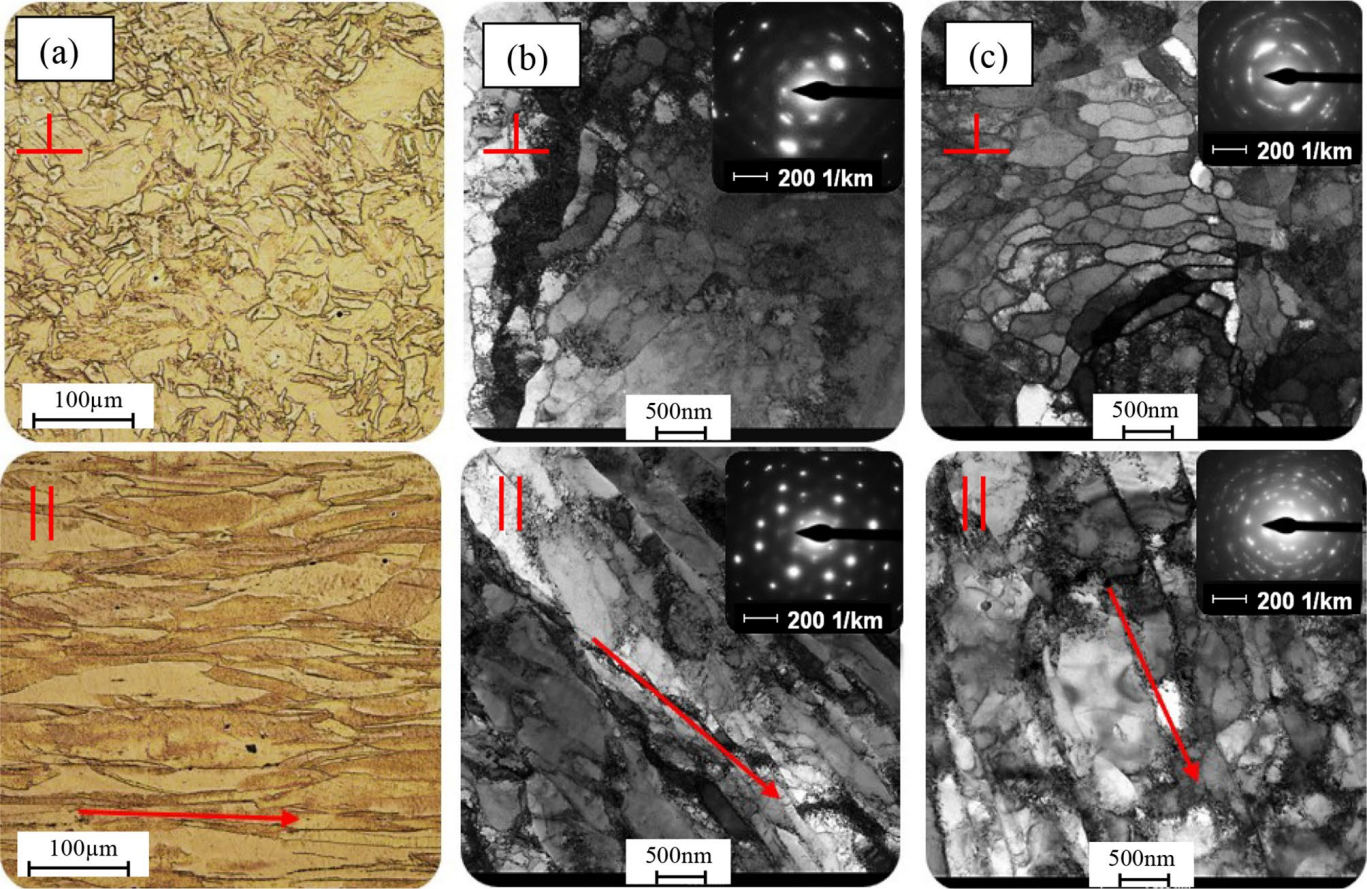
Microstructure of copper after single hydrostatic extrusion process: (**a**) *ɛ* = 1.12, (**b**) *ɛ* = 1.71, (**c**) *ɛ* = 2.53.  cross – section,  longitudinal section.

The direct factor affecting the changes in both mechanical and thermophysical properties of materials after Severe Plastic Deformation (SPD) processes is the evolution of the microstructure, which results in its significant fragmentation and generation of a large number of structural defects. In the below illustration, Fig. 3 the evolution of the copper microstructure after the one-stage HE process is presented.

The material in the initial state has a homogeneous isotropic microstructure with an average grain size of 35 µm. After the first stage of extrusion, which is common for both HE variants, we observed the microstructure of strongly deformed primary grains, Fig. 3a, elongated according to the direction of the rod extrusion, Fig. 3b. In subsequent one-step HE processes, the accumulation of defects and the formation of a dislocation microstructure was observed. An increase in the strain indicator to the level of 2.53 leads to pronounced thermal effects, recovery and dynamic recrystallisation, Fig. 3c. The average grain size at this stage was 377 nm. In the images of microstructures, a number of subgrains are observed in the areas where larger grains were formed. The lack of defects inside the subgrains may be linked to the intensive dynamic healing processes occurring during the plastic deformation. In all the tested specimens, at each strain stage, a clear anisotropic morphology of grains elongated in the direction of extrusion, with equiaxed grains visible in the cross-section, was observed.

The effects of heat-induced healing and recrystallisation processes in copper and other HE-processed materials have been repeatedly reported by the authors in previous works. An example is copper subjected to the HE process in cryogenic conditions^35^. Reducing the temperature in the process allowed to limit the adiabatic effects in the deformation zone and, consequently, limited the processes of recovery and dynamic recrystallisation taking place in the shaping mould. This allowed the refinement of the copper microstructure to the level of *d*_2_ = 320 nm and obtaining a strength equal to *UTS* = 490 MPa.

Another method to reduce undesirable thermal effects is to reduce the degree of deformation, and thus the adiabatic temperature during the HE process. This effect was achieved by using cumulative extrusion carried out in five stages with successively decreasing unit deformation in subsequent stages. As a result, a deformed material with accumulated true strain *ɛ*_*cum*_ = 3.89 was obtained when the adiabatic temperature was decreased at each stage of the process. This is evidenced by the analysis of the homologous temperature presented for both HE variants (Table 3). For the cumulative process, values *T/T*_*m*_ were almost identical for each of the stages and did not exceed 0.3.

**Figure 4:**
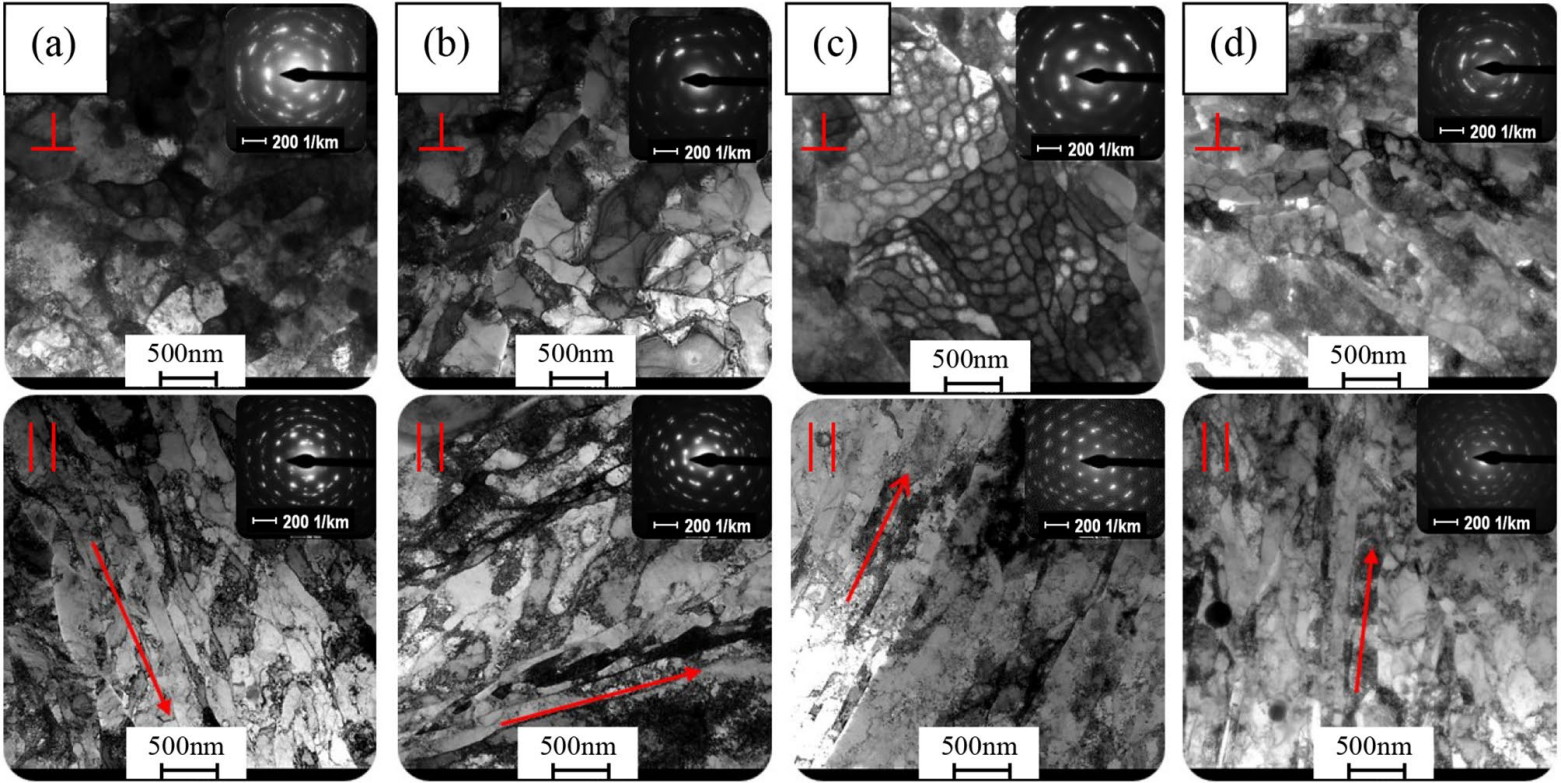
Microstructure of copper after cumulative hydrostatic extrusion process: (**a**) *ɛ* = 1.71, (**b**) *ɛ* = 2.53, (**c**) *ɛ* = 3.08, (**d**) ɛ = 3.89.  cross – section,  longitudinal section.

The illustration below (Fig. 4) presents the evolution of the copper microstructure after the cumulative HE process. Cumulative extrusion is much more effective, as evidenced by the fact that after the second stage of deformation with cumulative strain equal to ɛ = 1.7, (Fig. 4a) an ultrafine-grained microstructure with a large number of defects inside the grains can be observed. The average grain size at this stage was 300 nm. Further increase of strain to the level of ɛ = 2.53, (Fig. 4b) leads to dynamic healing processes accompanying plastic deformation, thanks to which a structure with clearly developed grains and a small number of defects is obtained. The average grain size is 370 nm. The next stage of the cumulative extrusion process shows clear thermal effects in the form of dynamic recrystallisation. This leads to the formation of sub-grain structures with clusters of grains free from defects, similar to the single extrusion. With the maximum degree of deformation equal to ɛ = 3.89, (Fig. 4d), the average grain size is 228 nm. The last HE process, with a relatively weak adiabatic heating effect related to not very high unit strain, results in effective refinement of the copper microstructure.

In the case of cumulative extrusion, we also observe a clear morphological anisotropy with characteristic elongated grains visible on the longitudinal sections of the tested specimens.

### Mechanical properties

**Figure 5:**
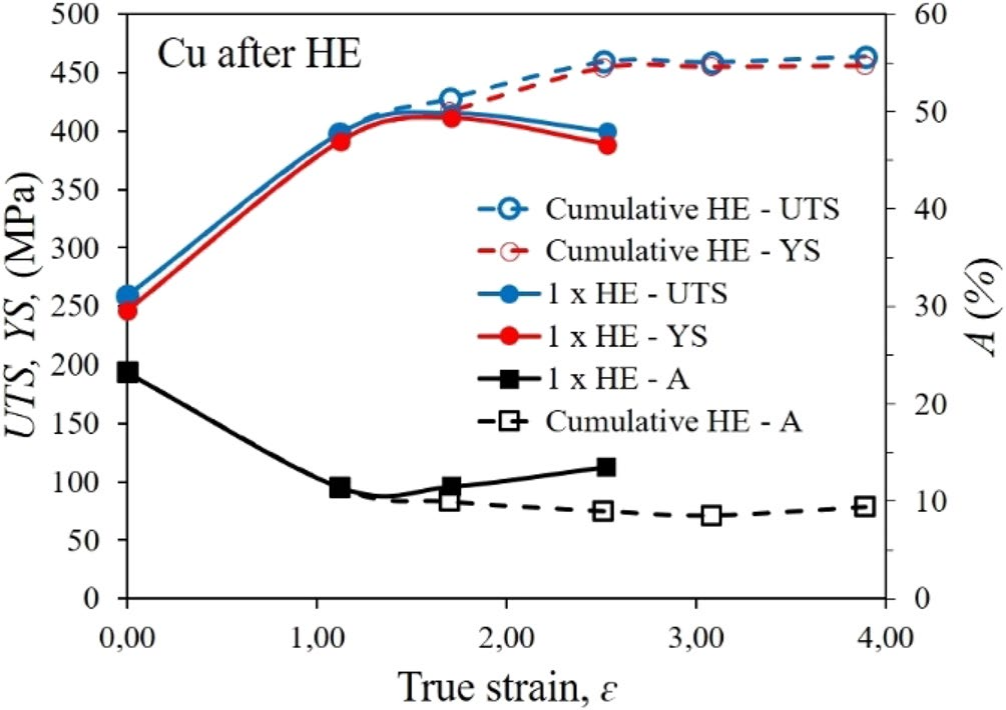
Dependence of *UTS* tensile strength and *YS* yield stress on true strain *ε* for copper after the single-step cold hydrostatic extrusions, and after the cumulative HE.

**Figure 6:**
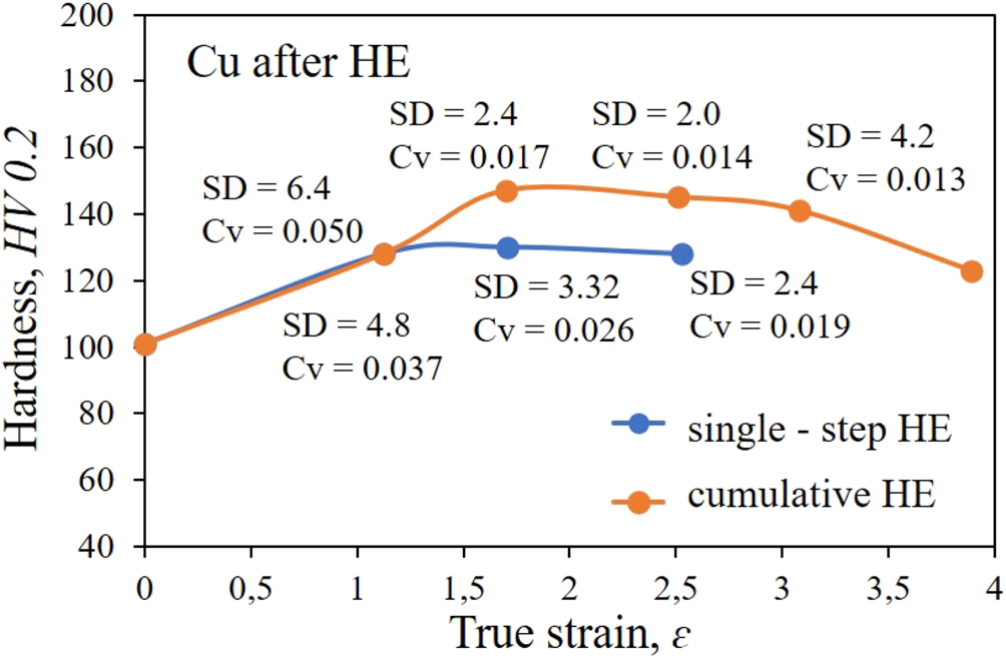
Dependence of hardness *HV*_2_*.*2 on true strain ε for copper after the single-step cold hydrostatic extrusions, and after the cumulative HE.

Figure 5 shows the strength properties of the tested copper after the HE processing and in the initial state. The results obtained in the static tensile test reflect clear differences in the structure of the material depending on the strain degree used. Single-stage extrusion with increasing unit strain in the range from ɛ = 1.12 to about ɛ = 2.53 indicates saturation with defects, strain increase and a significant influence of adiabatic effects weakening the strengthening effects. These effects result directly from the low SFE coefficient of ~ 55 mJ/m^2^, which makes Cu highly susceptible to recrystallisation and other thermally induced processes^36,37^. This phenomenon is reflected by the clear flattening of the characteristic with the unit strain increase, Fig. 5. Similar values of *UTS* and *YS* were obtained for all three degrees of unit deformation—400–415 MPa for *UTS* and 390 to 410 MPa for YS. These values are higher compared to the copper in the initial state by about 60% for *UTS* and by more than 65% for *YS.* Further strengthening of the material was possible thanks to the cumulative process, in which, as a result of 5 HE stages leading to the final diameter of the product equal to Ø10 mm, further increases in strength parameters were obtained. *UTS* = 464 MPa i *YS* = 456 MPa were obtained for the maximum cumulative deformation degree ɛ = 3.89. Similar effects were observed in hardness tests, and the results are shown in the below diagram, Fig. 6. The hardness measurements proved the large discrepancy of the distribution variation coefficient *Cv*, which is the marker of material homogeneity. This coefficient for copper after the single-stage HE process remains at a similar level after each stage of the process. After the cumulative process, along with the increase in strain, homogenisation of the material, and thus a decrease in the value of the *Cv* coefficient was observed. The coefficient reaches its lowest value of 0.013 after four stages of HE. After the last stage of HE, a decrease of the *Cv* coefficient value was observed, which may indicate the ongoing processes of healing and polygonisation of defects, reflected in the observed microstructure of the formed ultrafine grains.

Table 4 below shows the results of post-HE copper testing in relation to other significant plastic deformation (SPD) methods described in the literature.

**Table 4:** Comparison of mechanical properties and electrical conductivity of pure copper as described in this work with combinations of other processes presented in the professional literature.

Material	Processing	Sample size	True strain	Grain size, *d*_2_ (nm)	*UTS* (MPa)	*YS* (MPa)	*HV* 0.2	*IACS* (%)	References
Cu 99.7%	CBD^(1)^	210 µm	7.2	145	790	680	–	68.5	^38^
Cu 98.5%	ARB^(2)^	30 × 1 mm (wide x thickness)	50%	180	723	–	185	-	^39^
Cu 99.96%	ECAP^(3)^	10 mm	16	440	419.24	385	–	95.35	^40^
Cu 99.44%	ECAP + rolling	25 × 2 mm (wide x thickness)	–	610	320	–	112	81	^41^
Cu 99.96%	ECAP	10 mm	16	500	–	–	–	94.3	^42^
Cu 99.99%	ECAP	20 mm	4.62	–	388	331	–	–	^43^
Cu 99.97%	ECAP	10 mm	8	470	371	361	–	–	^44^
Cu 99.81%	ECAP	10 mm	8	520	361	343	–	–	^44^
Cu 99.95%	ECAP	10 × 10	12	440	–	–	125	–	^45^
Cu 99.95%	HE	10 mm	3.89	228	464	456	123	97.05	This work

^(1)^Continuous Bending-Drawing (CBD), ^(2)^Accumulative Roll-Bonding (ARB), ^(3)^Equal Chanell Angular Pressing (ECAP).

The presented results confirm the very high efficiency of the HE process compared to other SPD methods. Only the specimens produced using the Equal Channel Angular Pressing (ECAP) and HE processes allow for the production of material in a volume enabling the production of electrodes for EDM machining. In other cases, we are dealing with the production of thin sheets with very high strength parameters reaching *UTS* = 800 MPa, however, having dimensions that technically exclude their commercial use as electrodes for EDM machining.

The strength properties of Cu after the HE process obtained in this work exceed the remaining professional literature data both for the ECAP process^42–45^ and the combination of ECAP and rolling^41^. Despite the use of a higher number of plastic strain operations (higher strain degrees), they all generate significantly lower strength. This proves a much stronger effect of generating structural defects in the HE process compared to other methods, which is related to high deformation rates and thermal processes that simultaneously lead to effective refinement of the material microstructure. Plastic deformation rates in the HE method are two or three orders of magnitude higher than for the ECAP process, respectively, for HE $\dot{\varepsilon }$_HE_ ~ 10^3^ s^−1^ and for ECAP $\dot{\varepsilon }$_ECAP_ ~ 10^0^ s^−1^. The effective transformation of the work of the plastic deformation into the energy of accumulated defects was observed for many other metals subjected to the HE process^35,46,47^. Another positive effect observed for the HE process is the hydrostatic triaxial state of stress, which hinders the generation and propagation of cracks in the deformed material, which supports the preservation of the material consolidation state for a much higher range of plastic strain compared to other SPD methods.

### Electrical conductivity

**Figure 7:**
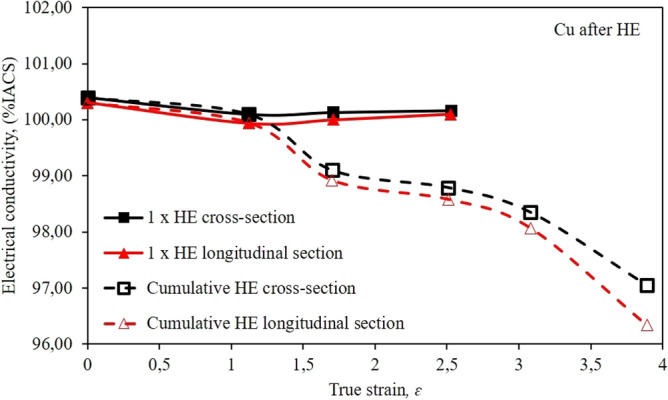
Dependence of electrical conductivity % IACS on true strain *ε* for copper after the single-step cold hydrostatic extrusions, and after the cumulative HE.

Due to the application potential of the tested copper after the HE process, the authors conducted an analysis of the electrical conductivity of samples after all deformation stages, both those the one-stage and cumulative HE processes, Fig. 7. Electrical conductivity measurements were taken on both the cross-section and the longitudinal section of the tested specimens. The obtained results clearly indicate the relation between the strain size and the electrical conductivity. The specimens subjected to deformation in the one-stage process with true strain in the range of *ɛ* = 1.12–2.53 have a similar value of electrical conductivity that equals to ~ 100% IACS, which corresponds to copper in the initial state. The almost linear nature of electrical conductivity as a function of strain results from the healing and recrystallisation processes that eliminate the effects of strain hardening, which is confirmed by the analysis of the copper microstructure after a single-stage process, Fig. 3.

During the process of cumulative extrusion, a decrease in electrical conductivity was observed along with the increase in strain, reaching a value of about 97% IACS after five HE stages. This phenomenon results from the much greater efficiency of the cumulative process. This allowed for the accumulation of an increasing number of structural defects in the tested material with the increase in strain, which constitutes a barrier to the flow of electrons. In both variants, a slightly lower electrical conductivity was also observed in the longitudinal section of the tested samples. A similar phenomenon of reducing the electrical conductivity due to the accumulation of structural defects in the material, which is an effective barrier to the flow of electrons, was also observed for methods of significant plastic deformations^38–45^. The Higuera-Cobos research can serve as an example, in which the authors subjected pure copper to plastic deformation using the ECAP method in 16 passes in B_c_ configuration. As a result, an ultrafine-grained microstructure was obtained with an average grain size of about 500 nm and an electrical conductivity of about 94% IACS^42^. The fact that the number of structural defects has a significant impact on the electrical conductivity of copper is also confirmed by the research carried out by Asiyeh Habibi^41^ and Mahla Afifeh^48^. The obtained values of electrical conductivity in both cases amounted to slightly more than 80% IACS and resulted from the use of cryogenic temperatures in the process of deformation. This made it possible to reduce the adiabatic effects and, consequently, to recrystallise the highly defective microstructure.

### Usability tests

Test electrodes for EDM machining were made of copper after the hydrostatic extrusion process. The electrodes had a diameter of 10 mm and a height of 60 mm. As part of the test, a workpiece made of steel WCL/1.2343/X37CRMOV5-1 was machined. The tests were carried out in two machining variants, i.e. rough machining and finish machining for reference purposes, electrodes made of non-deformed copper were subjected to similar tests.

**Figure 8:**
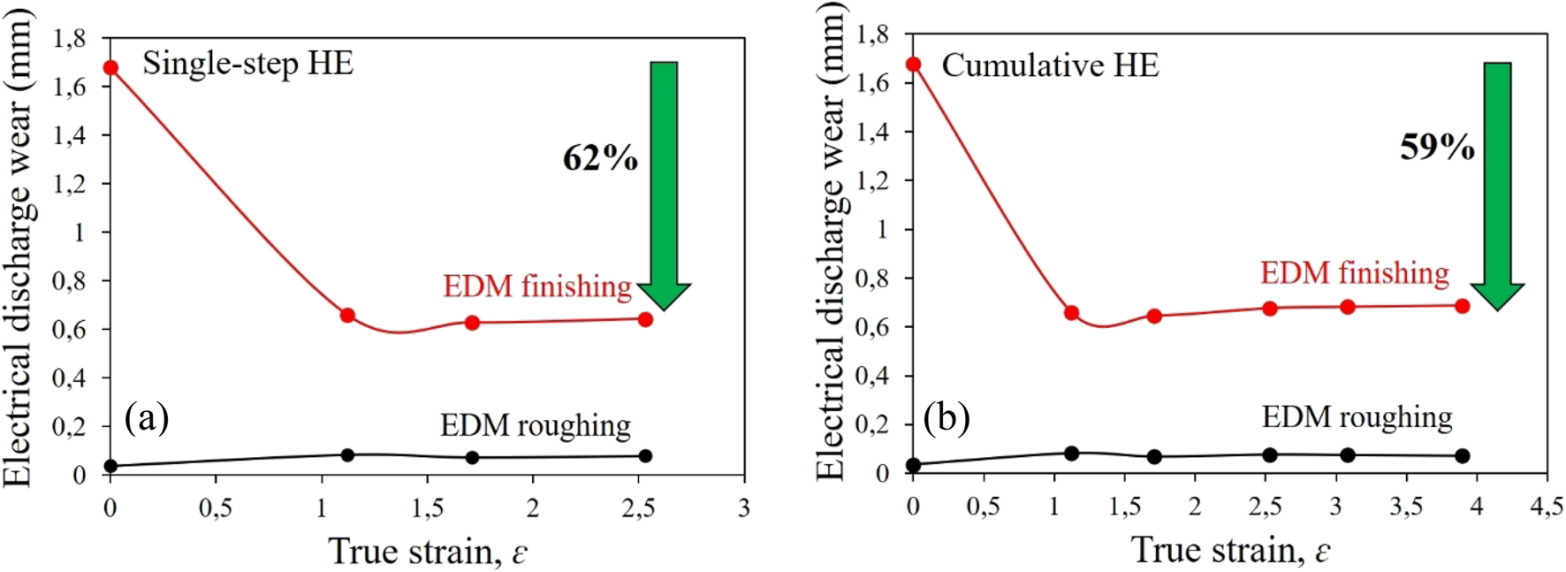
Evolution of electrical discharge wear in the function of true strain *ε* for copper electrode in EDM test: (**a**) single-step cold hydrostatic extrusions, (**b**) cumulative HE.

Figure 8, shows the dependence of copper erosion wear after the HE process as a function of actual deformation during the process of Electro Discharge Machining (rough machining, Fig. 8a and finish machining, Fig. 8b).

The results indicate that the refined ultra-fine-grained structure obtained in the deformation process has a positive effect on the wear of the tested copper electrode, which is particularly visible in the process of finish machining. This is due to the fact that the finish machining using EDM process has much lower machining parameter values, such as operating amperage, *I*_*r*_ (Table 2). These parameters significantly affect the temperature in the erosion zone. As a result, the electrodes operating under intense load, i.e. in rough machining, are degraded due to the processes of recovery and recrystallisation. The result is erosion wear almost identical to that occurring in non-deformed copper. However, in the case of finish machining where the machining parameters are much lower (working amperage, *I*_*r*_ = 3 A), a clearly lower operational wear of about 0.7 mm was observed, which is a decrease of over 60% compared to the electrode made of undeformed copper, Fig. 8a. An almost identical effect is observed for the electrodes made of copper after the cumulative HE process, Fig. 8b. In this case, a slight increase in electrode wear with increasing deformation was observed, but ultimately, for the sample after the HE process with the largest cumulative deformation of *ɛ* = 3.89, the operational wear turned out to be lower than that of non-deformed material by almost 60%. In the case of electrodes made of copper subjected to the cumulative process, the effect of a slight increase in wear with an increase in deformation is caused by a decrease of electrical conductivity after the HE process.

There is no information in the professional literature on EDM wear tests of electrodes produced in unconventional plastic deformation processes with large deformations, however, the authors of this paper proved the advantage of the HE process over other methods in similar application solutions. An example is the research carried out by Kulczyk et al., where the authors conducted wear tests of electrodes used for the resistance welding process made of alloy copper CuCr1Zr after the HE process, additionally strengthened by precipitation in the aging process^31^. The test performed proved an over six-fold reduction in electrode wear after the HE process compared to commercial electrodes. In addition, for comparative purposes, electrodes made of copper produced by another unconventional method, i.e. ECAP, were also subjected to wear tests. In this case, the electrodes turned out to be only slightly better than commercial ones, despite the copper microstructure was clearly refined. The authors attribute these effects to the characteristic morphology of grains elongated in the direction of extrusion.

**Figure 9:**
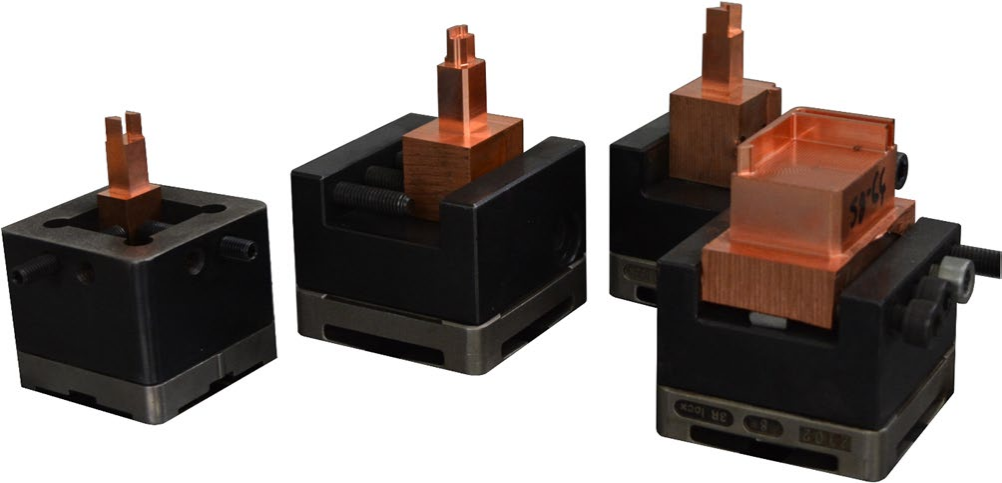
Prototype electrodes for the EDM machining processes.

Based on the results of physical and operational tests of copper after the HE process, the authors designed and manufactured prototype electrodes for the EDM machining processes (Fig. 9). The electrodes will be verified in the further production of injection moulds.

## Summary

This paper presents a study of the effect of microstructure refinement of pure copper after the hydrostatic extrusion process on EDM properties. The presented results confirmed the effectiveness of the hydrostatic extrusion process as one of the methods that allow to give new and better properties to materials giving a technological advantage in the previously used machining processes.

Conclusions of the obtained research results are as follows:

1. HE method allows effective microstructure refinement of copper. The average grain size after the HE process with a cumulative true strain of ɛ = 3.89 was, d2 = 228 nm.
2. As a result of microstructure refinement in the tested copper, a significant increase in strength properties compared to the material in the initial state was obtained equal for YS by 85% and UTS by 80% with a slight decrease in electrical conductivity equal to 3% IACS compared to the material in the initial state.
3. Conducted EDM wear tests showed more than 60% lower wear of electrodes after the HE process compared to the commercial material with the best results obtained for EDM finishing characterized by low values of machining parameters in the form of working current, Ir, which do not have a destructive effect on the fragmented high-energy microstructure refinement obtained in the HE process susceptible to heat-induced healing and recrystallization processes.
4. Optimizing the EDM process to include the use of a new group of electrode materials will significantly improve process efficiency. Less EDM wear means fewer electrodes needed in the EDM process and shorter process time itself.
5. The energy-intensive aspect is also not insignificant. Lower EDM consumption will allow for significantly shorter machining, thus reducing energy requirements.

## Data Availability

The datasets used and/or analyzed during the current study available from the corresponding author on reasonable request.
